# Preclinical Evaluation of Dual NOP/Mu Partial Agonists for Methamphetamine Use Disorder

**DOI:** 10.3390/ph19071119

**Published:** 2026-07-20

**Authors:** Thamires Righi, Gilles Zribi, Sebastian Garcia, Stephen M. Husbands, Lawrence Toll, Andrea Cippitelli

**Affiliations:** 1 Biomedical Science Department, Charles E. Schmidt College of Medicine, Florida Atlantic University, Boca Raton, FL 33431, USA; 2 Department of Pharmacy and Pharmacology, University of Bath, Bath BA2 7AY, UK; 3 Stiles-Nicholson Brain Institute, Florida Atlantic University, Jupiter, FL 33458, USA

**Keywords:** methamphetamine, food, choice, self-administration, reinstatement, reward, NOP receptor, mu opioid receptor, seeking, priming, cues

## Abstract

**Background/Objectives:** Methamphetamine (meth) use disorder (MUD) continues to pose a significant public health challenge, and there are currently no FDA-approved pharmacological treatments available. Compounds that simultaneously activate nociceptin opioid peptide (NOP) and mu opioid receptors offer a promising therapeutic approach for substance use disorders, including psychostimulant addiction. **Methods:** We evaluated two mixed NOP/mu receptor agonists for their ability to reduce meth intake using a translational drug-versus-food choice self-administration paradigm in male and female Sprague–Dawley rats. In addition, both compounds were tested in female rats for their effects on cue- and priming-induced reinstatement of meth-seeking behavior, established preclinical models of relapse. **Results:** PPL-138 and PPL-143 are structurally related bifunctional NOP/mu partial agonists that exhibit greater efficacy at NOP than at mu receptors, with PPL-143 showing the highest NOP efficacy. In behavioral assays, both compounds significantly reduced meth self-administration in male rats and exhibited comparable potency in this effect. In female rats, however, PPL-143 was less effective than PPL-138 in reducing meth intake. In reinstatement models, PPL-138, but not PPL-143, attenuated prime-induced reinstatement of meth seeking, whereas both compounds produced comparable increases in meth seeking in the cue-induced reinstatement paradigm. Additional findings indicated that PPL-138 enhanced meth-induced locomotor activation. **Conclusions:** The findings support mixed mu/NOP partial agonists as a promising pharmacological strategy for treating MUD, while also indicating a potential for increased drug seeking under certain conditions. Notably, enhancing the NOP component does not appear to meaningfully improve either the safety profile or the anti-meth efficacy of these bifunctional ligands.

## 1. Introduction

Effective pharmacotherapies are available for opioid, alcohol, and nicotine use disorders, and extensive clinical efforts have evaluated candidate treatments for cocaine dependence. In contrast, progress in developing medications for methamphetamine (meth) use disorder (MUD) has been comparatively limited. MUD is an increasingly significant public health concern, with epidemiological evidence indicating both rising prevalence and substantial clinical burden. Over time, meth use and related disorders have increased markedly; past-year use among adults rose significantly between 2016 and 2019, particularly among individuals aged 26 years and older [1]. More recent national survey data indicate that approximately 2.4 million people aged 12 years or older in the United States reported meth use in the past year, representing about 0.8% of the population [2]. The consequences of meth use are considerable with amphetamine-type stimulants implicated in approximately 15% of all drug overdose deaths [3,4].

Meth, a fully synthetic compound, acts as a substrate for both vesicular and plasma membrane monoamine transporters, including dopamine (DA), serotonin (5-HT) and noradrenaline (NA) transporters. This process results in the reverse transport of DA, 5-HT, and NA into the synapse, which then stimulate postsynaptic monoamine receptors [5]. Meth induces far larger extracellular levels of DA than cocaine, and the effects of meth last much longer, with a “high” lasting for hours [6]. Meth also attenuates monoamine metabolism by inhibiting monoamine oxidase [7], further contributing to the buildup of excess monoamines in the synapse. Other stimulants such as bupropion, methylphenidate, and modafinil have been somewhat successful in reducing meth self-administration in animals and people [8]. Compounds in clinical trials include entacapone, an inhibitor of catechol-O-methyltransferase, which can increase DA availability by reducing DA metabolism. The glial attenuator ibudilast is also in clinical trials, as is IXT-m200, a monoclonal antibody [9,10]. The combination of naltrexone and bupropion is currently considered the most promising pharmacological option for MUD [11]. Unfortunately, however, none of these compounds has shown sufficient success to be approved by the FDA for the treatment of MUD, and behavioral therapies (e.g., cognitive–behavioral therapy and motivational incentives) remain the most effective treatments for meth addiction [12].

The opioid receptor family comprises four principal subtypes: mu, delta, kappa, and nociceptin/orphanin FQ (NOP), all highly expressed in brain regions and neural circuits implicated in reward and substance use disorders [13,14]. Most clinically used opioid analgesics act as mu receptor agonists, and activation of this receptor is strongly associated with euphoria and the development of addiction. In contrast, activation of the NOP receptor has been proposed to modulate or counterbalance mu receptor signaling, thereby potentially reducing abuse liability [15,16,17]. Consistently, both the endogenous ligand nociceptin/orphanin FQ (N/OFQ) and small-molecule NOP agonists have been shown to block the acquisition of conditioned place preference (CPP) induced by morphine, cocaine, amphetamine, and alcohol [17,18,19,20,21,22]. N/OFQ also suppresses basal hedonic tone and attenuates acute reward responses to meth [23]. These observations are further supported by studies demonstrating that NOP agonists attenuate morphine- and cocaine-induced increases in extracellular DA within the nucleus accumbens [24,25,26,27]. In line with these findings, we have shown that the NOP receptor agonist AT-312 reduces cocaine self-administration [28], supporting the idea that compounds with an appropriate balance of NOP and mu receptor activity can attenuate psychostimulant use. This concept is further supported by compounds such as cebranopadol, a dual mu/NOP receptor agonist that retains robust analgesic efficacy while exhibiting relatively low abuse potential and reported anti-cocaine effects [29,30,31,32,33].

Building on this rationale, we have developed a series of novel analgesics that function as partial agonists at both NOP and mu opioid receptors, while acting as antagonists at kappa opioid receptors, a pharmacological profile similar to that of buprenorphine. However, despite its clinical utility, buprenorphine retains measurable abuse potential [34]. To address this limitation, we have designed compounds with greater affinity and efficacy at the NOP receptor while maintaining only minimal partial agonist activity at the mu receptor [35]. This approach is intended to enhance their effectiveness in treating substance use disorders while reducing abuse liability. One such compound, BU10038 (now PPL-138), exhibits highly potent analgesic effects, approximately 100-fold greater than morphine, along with a reduced side-effect profile in non-human primates [36]. Notably, it has also been shown to decrease cocaine self-administration in rats [37], as well as escalated alcohol self-administration in rats with a history of traumatic-like stress exposure [38], further supporting the therapeutic promise of NOP-directed ligands.

In the present study, we hypothesized that bifunctional NOP/mu partial agonists would attenuate behavioral measures relevant to MUD, and that compounds with enhanced NOP receptor activity would produce greater efficacy. To evaluate this hypothesis, we employed a translational model incorporating meth-versus-food choice paradigms and two complementary reinstatement procedures. This approach enables assessment of these compounds across two key features of MUD: ongoing drug taking despite the availability of a nondrug alternative and relapse-like behavior.

## 2. Results

### 2.1. Effects of PPL-138 Treatment on Meth-Vs-Food Choice in Male Rats

**Figure 1:**
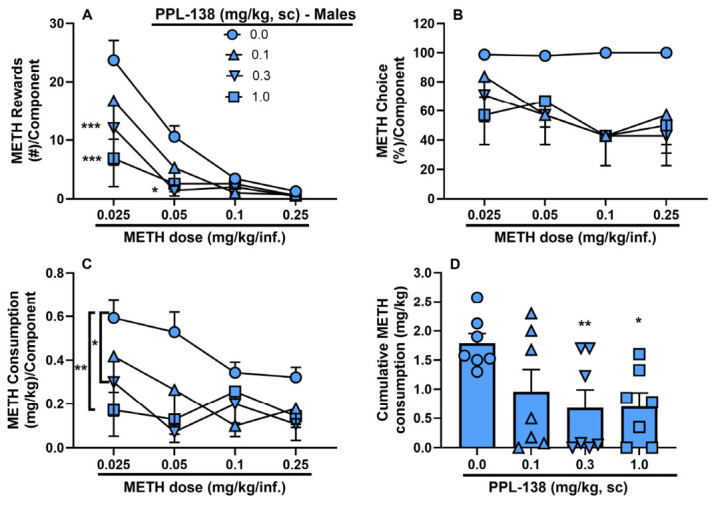
Effects of PPL-138 on meth-vs-food choice self-administration paradigm in male rats. (**A**) Number (#) of methamphetamine (meth) rewards earned per component as the meth dose (mg/kg) increased across successive 29 min components (low to high), and the effects of subcutaneous (sc) PPL-138 at 0.1, 0.3, and 1.0 mg/kg on meth rewards within each component. (**B**) Percent (%) meth choice as a function of meth dose (mg/kg) across components, and the effects of PPL-138 at all doses on choice between meth and food pellets. (**C**) Meth intake (mg/kg) per component as a function of increasing dose, and the effects of PPL-138. (**D**) Cumulative meth intake (mg/kg) across components and the effects of each PPL-138 dose. All data are presented as mean ± SEM (n = 7 male rats). Asterisks indicate significant differences from vehicle (0.0 mg/kg PPL-138): * *p* < 0.05, ** *p* < 0.01, *** *p* < 0.001.

An analysis of meth rewards revealed a significant “component” × “PPL-138 dose” interaction [*F*(9,54) = 4.5, *p* < 0.001], indicating that the effects of PPL-138 on meth self-administration were dose-dependent. This interaction likely reflected baseline differences in responding, as rats exhibited high response rates at lower meth doses and substantially lower response rates at higher doses. Post hoc analyses showed that PPL-138 significantly reduced self-administration of lower meth doses (0.025 mg/kg/infusion: *p* < 0.001 at 0.3 and 1 mg/kg; 0.05 mg/kg/infusion: *p* < 0.05 at 0.3 mg/kg; Figure 1A). Analysis of meth choice after transforming the data to area-under-the-curve (AUC) indicated an overall trend toward reduced meth preference, although this effect did not reach statistical significance [*F*(3,18) = 2.4, *p* = 0.09, (Figure 1B)]. Analysis of meth consumption per component revealed a significant main effect of PPL-138 dose [*F*(3,18) = 5.7, *p* < 0.01]. Post hoc comparisons indicated that the 0.3 mg/kg (*p* < 0.01) and 1 mg/kg (*p* < 0.05) doses differed significantly from the vehicle (Figure 1C). Cumulative meth consumption across components and PPL-138 treatment conditions is shown in Figure 1D.

### 2.2. Effects of PPL-138 Treatment on Meth-Vs-Food Choice in Female Rats

**Figure 2:**
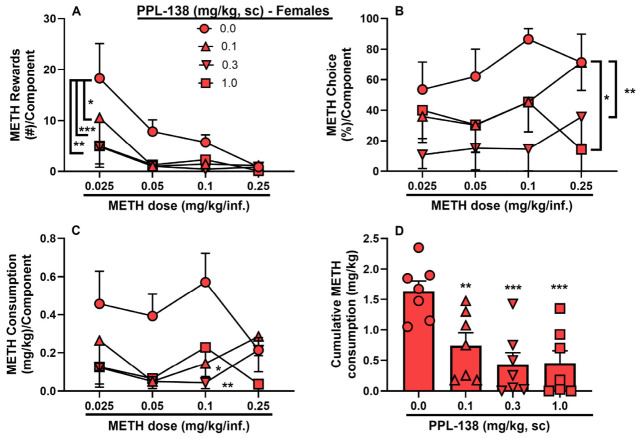
Effects of PPL-138 on meth-vs-food choice self-administration paradigm in female rats. (**A**) Number (#) of methamphetamine (meth) rewards earned per component as the meth dose (mg/kg) increased across successive 29 min components (low to high), and the effects of subcutaneous (sc) PPL-138 at 0.1, 0.3, and 1.0 mg/kg on meth rewards within each component. (**B**) Percent (%) meth choice as a function of meth dose (mg/kg) across components, and the effects of PPL-138 at all doses on choice between meth and food pellets. (**C**) Meth intake (mg/kg) per component as a function of increasing dose, and the effects of PPL-138. (**D**) Cumulative meth intake (mg/kg) across components and the effects of each PPL-138 dose. All data are presented as mean ± SEM (n = 7 female rats). Asterisks indicate significant differences from vehicle (0.0 mg/kg PPL-138): * *p* < 0.05, ** *p* < 0.01, *** *p* < 0.001.

Analysis of meth reward revealed a significant main effect of “PPL-138 dose” [*F*(3,18) = 9.6, *p* < 0.001]. There was also a marginally significant main effect of “component” [*F*(3,18) = 3.1, *p* = 0.05], but no significant “component” × “PPL-138 dose” interaction [*F*(9,54) = 1.7, *p* = 0.09]. Post hoc analysis conducted across the main PPL-138 treatment effect indicated that all three doses significantly reduced meth self-administration (0.1 mg/kg: *p* < 0.05; 0.3 mg/kg: *p* < 0.001; 1.0 mg/kg: *p* < 0.01; Figure 2A). Analysis of METH choice demonstrated that PPL-138 significantly reduced preference for meth when food was concurrently available [*F*(3,18) = 6.0, *p* < 0.01; AUC transformed data [*F*(3,18) = 6.2, *p* < 0.01]. Post hoc comparisons indicated that doses of 0.3 mg/kg (*p* < 0.01) and 1.0 mg/kg (*p* < 0.05) significantly decreased meth choice (Figure 2B). Analysis of meth consumption per component revealed a significant “component” × “PPL-138 dose” interaction [*F*(9,54) = 2.1, *p* < 0.05] (post hoc for 0.01 mg/kg/infusion: *p* < 0.01 at 0.3 mg/kg and *p* < 0.05 at 1 mg/kg, Figure 2C). Cumulative meth consumption was reduced by PPL-138 treatment [*F*(3,18) = 14.0, *p* < 0.001], at all doses examined (0.1 mg/kg: *p* < 0.01; 0.3 and 1 mg/kg: *p* < 0.001, Figure 2D).

### 2.3. Effects of PPL-143 Treatment on Meth-Vs-Food Choice in Male Rats

**Figure 3:**
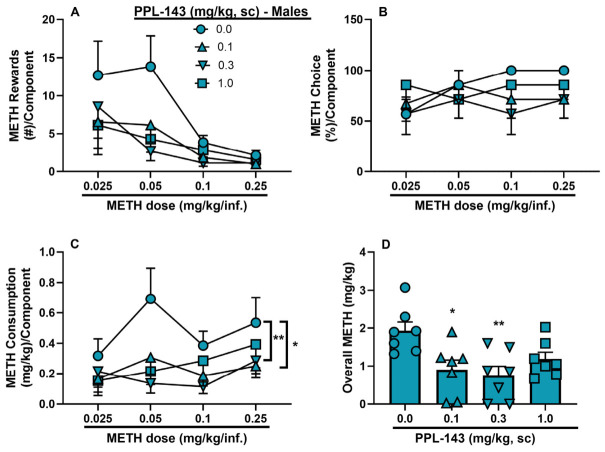
Effects of PPL-143 on meth-vs-food choice self-administration paradigm in male rats. (**A**) Number (#) of methamphetamine (meth) rewards earned per component as the meth dose (mg/kg) increased across successive 29 min components (low to high), and the effects of subcutaneous (sc) PPL-143 at 0.1, 0.3, and 1.0 mg/kg on meth rewards within each component. (**B**) Percent (%) meth choice as a function of meth dose (mg/kg) across components, and the effects of PPL-143 at all doses on choice between meth and food pellets. (**C**) Meth intake (mg/kg) per component as a function of increasing dose, and the effects of PPL-143. (**D**) Cumulative meth intake (mg/kg) across components and the effects of each PPL-143 dose. All data are presented as mean ± SEM (n = 7 male rats). Asterisks indicate significant differences from vehicle (0.0 mg/kg PPL-143): * *p* < 0.05, ** *p* < 0.01.

Analysis of meth reward revealed a significant main effect of “PPL-143 dose” [*F*(3,18) = 3.5, *p* < 0.05] and “component” [*F*(3,18) = 5.5, *p* < 0.01], with no significant “component” × “PPL-143 dose” interaction [*F*(9,54) = 1.1, *p* = 0.35]. Post hoc analysis conducted across the main PPL-143 treatment effect indicated that all three doses led to a trend to reduce METH self-administration (0.1 mg/kg: *p* = 0.09; 0.3 mg/kg: *p* = 0.05; 1.0 mg/kg: *p* = 0.07; Figure 3A). Analysis of meth choice after transforming the data to AUC indicated that meth preference remained unchanged following PPL-143 treatment [*F*(3,18) = 0.9, *p* = 0.44, (Figure 3B)]. Analysis of meth consumption per component revealed a significant main effect of PPL-143 dose [*F*(3,18) = 5.7, *p* < 0.01] accompanied by not significant “effect of PPL-143” × “component” interaction [*F*(9,54) = 0.8, *p* = 0.6]. Post hoc comparisons of the main PPL-143 treatment effect indicated that the 0.1 mg/kg (*p* < 0.05) and 0.3 mg/kg (*p* < 0.01) doses differed significantly from the vehicle, whereas the 1 mg/kg dose had a trend toward a reduction (*p* = 0.05, Figure 3C). Cumulative meth consumption across components and PPL-143 treatment conditions is presented in Figure 3D.

### 2.4. Effects of PPL-143 Treatment on Meth-Vs-Food Choice in Female Rats

**Figure 4:**
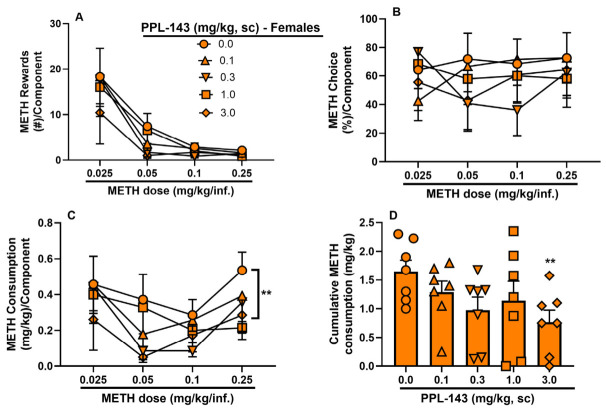
Effects of PPL-143 on meth-vs-food choice self-administration paradigm in female rats. (**A**) Number (#) of methamphetamine (meth) rewards earned per component as the meth dose (mg/kg) increased across successive 29 min components (low to high), and the effects of subcutaneous (sc) PPL-143 at 0.1, 0.3, 1.0 and 3.0 mg/kg on meth rewards within each component. (**B**) Percent (%) meth choice as a function of meth dose (mg/kg) across components, and the effects of PPL-143 at all doses on choice between meth and food pellets. (**C**) Meth intake (mg/kg) per component as a function of increasing dose, and the effects of PPL-143. (**D**) Cumulative meth intake (mg/kg) across components and the effects of each PPL-143 dose. All data are presented as mean ± SEM (n = 7 male rats). Asterisks indicate significant differences from vehicle (0.0 mg/kg PPL-143): ** *p* < 0.01.

Analysis of meth reward revealed a significant main effect of component [*F*(3,18) = 6.0, *p* < 0.01]. In contrast, there was no significant main effect of PPL-143 dose [*F*(4,24) = 2.5, *p* = 0.07], even when the highest dose (3.0 mg/kg) was included. Moreover, the component × PPL-143 dose interaction was not significant [*F*(12,72) = 1.0, *p* = 0.48, (Figure 4A)]. Similarly, analysis of meth choice following PPL-143 treatment revealed no significant effect [*F*(4,24) = 0.5, *p* = 0.73; AUC transformed data [*F*(4,24) = 0.8, *p* = 0.5 (Figure 4B)]. In contrast, analysis of meth consumption per component showed a significant main effect of PPL-143 dose [*F*(4,24) = 4.0, *p* < 0.05], with no significant PPL-143 dose × component interaction [*F*(12,72) = 0.8, *p* = 0.70]. Post hoc comparisons for the main effect of PPL-143 dose indicated that only the 3.0 mg/kg dose differed significantly from the vehicle (*p* < 0.01; Figure 4C). Cumulative meth consumption across components and PPL-143 treatment conditions is shown in Figure 4D.

### 2.5. Effects of PPL-138 Treatment on Meth Prime- and Cue-Induced Reinstatement of Drug Seeking in Female Rats

**Figure 5:**
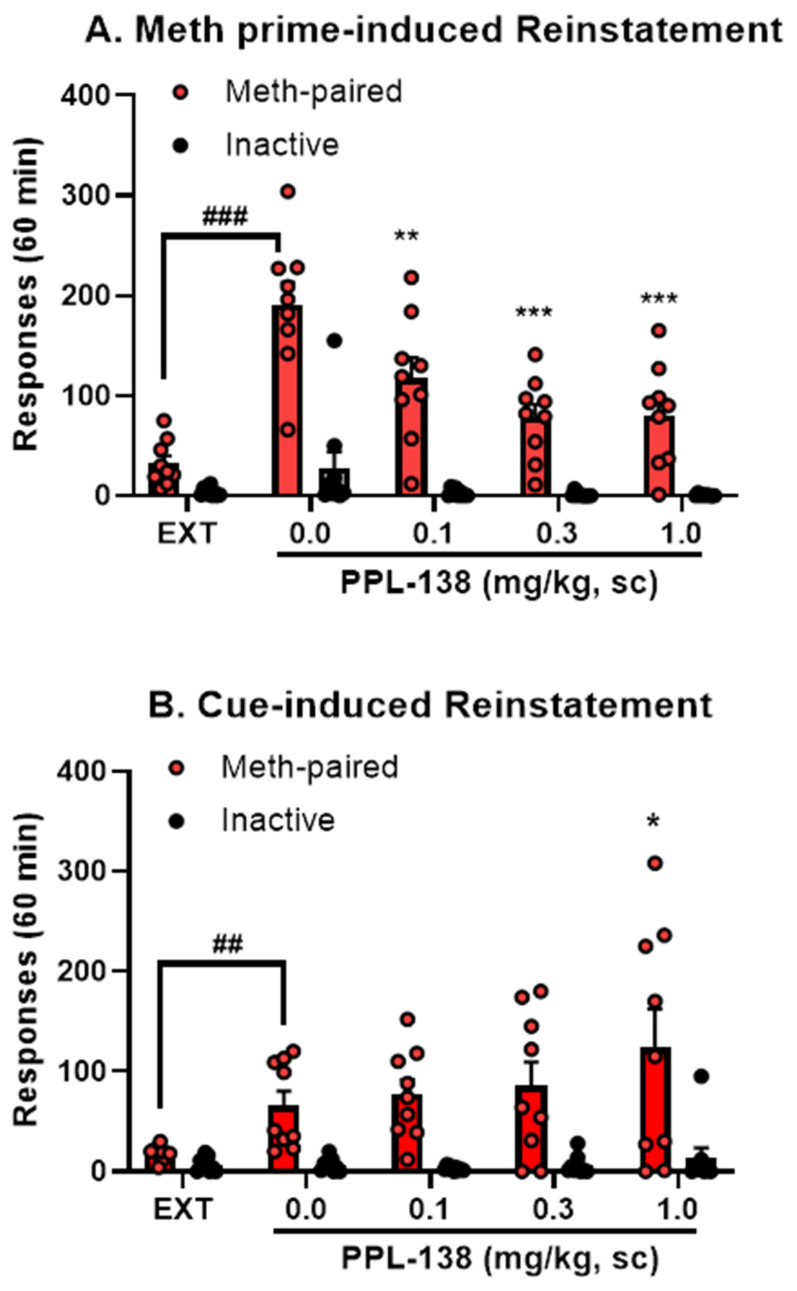
Effects of PPL-138 on methamphetamine (meth) priming-induced and cue-induced reinstatement models in female rats. (**A**) Reinstatement responding during a 60 min session, relative to a 60 min extinction (EXT) baseline, following an intraperitoneal (ip) meth prime (0.5 mg/kg, administered 15 min before the session). Effects of subcutaneous (sc) PPL-138 (0.1, 0.3, or 1.0 mg/kg, administered 20 min prior to the meth injection) on reinstatement responding are shown. PPL-138 attenuated meth prime-induced reinstatement of drug seeking. (**B**) Reinstatement responding during a 60 min session, relative to a 60 min EXT baseline, following exposure to previously meth-associated cues (light + tone). Effects of PPL-138 at all doses on reinstatement responding are shown. PPL-138 enhanced cue-induced reinstatement of meth seeking. All data are presented as mean ± SEM (n = 9 female rats per reinstatement modality). Asterisks denote significant differences from vehicle (0.0 mg/kg PPL-138): * *p* < 0.05, ** *p* < 0.01, *** *p* < 0.001. Pound signs denote significant differences from EXT: ^##^ *p* < 0.01, ^###^ *p* < 0.001. Red dots and bars denote meth-associated lever responses. Black dots and bars denote inactive lever responses.

As shown in Figure 5A, meth produced a robust reinstatement of drug-seeking behavior [t(8) = −7.2, *p* < 0.001]. Reinstatement was significantly attenuated by pretreatment with PPL-138 [*F*(3,24) = 16.7; *p* < 0.001]. Post hoc analyses revealed significant reductions at all three PPL-138 doses tested (0.1 mg/kg: *p* < 0.01, 0.3 and 1.0 mg/kg: *p* < 0.001). Responses on the inactive lever were not significantly altered by the meth prime [t(8) = −1.5, *p* = 0.2] and were also unaffected by PPL-138 treatment [*F*(3,24) = 2.6; *p* = 0.07]. Cues previously associated with meth delivery also produced a robust reinstatement of drug-seeking behavior [t(8) = −3.6, *p* < 0.01]; however, unexpectedly, PPL-138 treatment significantly increased rather than reduced drug seeking [*F*(3,24) = 3.2; *p* < 0.05]. On post hoc analysis, PPL-138 further increased reinstatement responses at 1.0 mg/kg (*p* < 0.05, Figure 5B). Responses on the inactive lever were not significantly altered by the meth-associated cues [t(8) = 1.1, *p* = 0.3] and were also unaffected by PPL-138 treatment [*F*(3,24) = 1.0; *p* = 0.4].

### 2.6. Effects of PPL-143 Treatment on Meth Prime- and Cue-Induced Reinstatement of Drug Seeking in Female Rats

**Figure 6:**
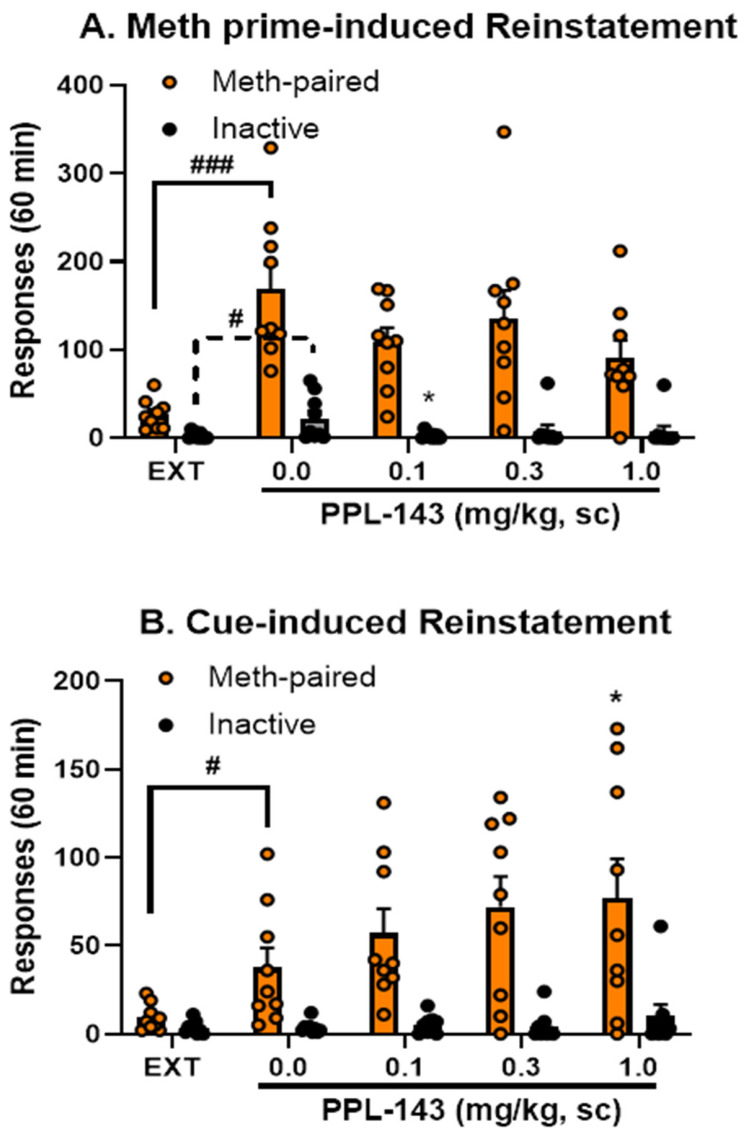
Effects of PPL-143 on methamphetamine (meth) priming-induced and cue-induced reinstatement models in female rats. (**A**) Reinstatement responding during a 60 min session, relative to a 60 min extinction (EXT) baseline, following an intraperitoneal (ip) meth prime (0.5 mg/kg, administered 15 min before the session). Effects of subcutaneous (sc) PPL-143 (0.1, 0.3, or 1.0 mg/kg, administered 20 min prior to the meth injection) on reinstatement responding are shown. PPL-143 did not alter meth prime-induced reinstatement of drug seeking. (**B**) Reinstatement responding during a 60 min session, relative to a 60 min EXT baseline, following exposure to previously meth-associated cues (light + tone). Effects of PPL-143 at all doses on reinstatement responding are shown. PPL-143 enhanced cue-induced reinstatement of meth seeking. All data are presented as mean ± SEM (n = 9 female rats per reinstatement modality). Asterisks denote significant differences from vehicle (0.0 mg/kg PPL-143): * *p* < 0.05. Pound signs denote significant differences from EXT: ^#^ *p* < 0.05, ^###^ *p* < 0.001. Orange dots and bars denote meth-associated lever responses. Black dots and bars denote inactive lever responses.

Even after treatment with PPL-138, meth continued to trigger robust reinstatement of drug-seeking behavior [t(8) = −5.3, *p* < 0.001]. However, as shown in Figure 6A, PPL-143 treatment did not significantly attenuate meth-primed reinstatement [*F*(3,24) = 2.7; *p* = 0.065]. Responses on the inactive lever were also significantly increased following the meth challenge [t(8) = −2.6, *p* < 0.05] and were reduced by PPL-143 treatment [*F*(3,24) = 4.2; *p* < 0.05], with a significant reduction observed at 0.1 mg/kg (*p* < 0.05). Cues previously associated with meth delivery also produced a robust reinstatement of drug-seeking behavior [t(8) = −2.7, *p* < 0.05]. PPL-143, as previously seen for PPL-138, significantly increased cue-induced drug seeking [*F*(3,24) = 3.5; *p* < 0.05]. On post hoc analysis, PPL-143 further increased reinstatement responses at 1.0 mg/kg (*p* < 0.05, Figure 6B). Responses on the inactive lever were not significantly altered by the meth-associated cues [t(8) = −0.2, *p* = 0.9] and were also unaffected by PPL-143 treatment [*F*(3,24) = 1.4; *p* = 0.3].

### 2.7. Effects of Combined PPL-138 and Meth Treatment on Locomotor Activity in Female Rats

**Figure 7:**
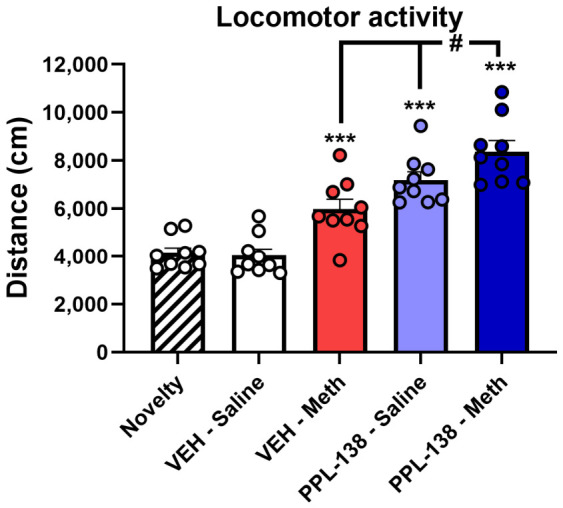
Effect of the combined PPL-138 and methamphetamine (meth) treatment on locomotor activity in female rats. Distance traveled (cm) by female rats during a 10 min habituation (novelty exposure) session and across four subsequent treatment sessions (10 min) in which VEH-Saline, VEH-meth, PPL-138-Saline, and PPL-138-meth were administered using a counterbalanced Latin square, within-subject design. PPL-138 (1 mg/kg) or vehicle was injected subcutaneously 20 min prior to meth injection, which occurred 15 min prior to open field. PPL-138 potentiated meth-induced locomotor activation. Data are presented as mean ± SEM (n = 9 female rats). Asterisks indicate significant differences relative to VEH-Saline controls (*** *p* < 0.001). Pound symbol indicates significant differences relative to both VEH-meth and PPL-138-Saline groups (^#^ *p* < 0.05).

Combined treatment with PPL-138 and meth significantly increased locomotor activity in female rats [*F* (3,24) = 54.3, *p* < 0.001]. Post hoc analysis revealed that both meth alone and PPL-138 alone significantly increased locomotor activity compared with controls (*p* < 0.001 for both). In addition, the combined treatment produced a significantly greater increase in locomotor activity than either treatment alone (*p* < 0.05 vs. both, Figure 7).

## 3. Discussion

PPL-138 and PPL-143 were developed as buprenorphine analogs with enhanced NOP receptor activity while preserving the favorable structural, pharmacokinetic, and opioid receptor properties of buprenorphine [35,36]. Both compounds exhibited greater NOP receptor affinity and efficacy than buprenorphine, with PPL-143 showing the highest NOP efficacy. Importantly, they retained high-affinity, low-efficacy interactions at mu opioid receptors and antagonism at kappa opioid receptors, a pharmacological profile that may be beneficial for the treatment of substance use disorders, including MUD.

The present study evaluated the effects of PPL-138 and PPL-143 on meth reinforcement, choice behavior, and relapse-like responding using a meth-versus-food choice paradigm and reinstatement models in male and female rats. Several key findings emerge: (i) PPL-138 robustly reduced meth self-administration, consumption and choice, with greater efficacy in females than males; (ii) PPL-143 exhibited weaker and less consistent effects than PPL-138, particularly in female rats, with modest reductions in consumption and limited impact on choice or reinstatement; (iii) both compounds failed to reduce, and in fact potentiated, cue-induced reinstatement; and (iv) PPL-138 enhanced meth-induced locomotor activation, which may account for the observed cue-induced meth seeking.

PPL-138 produced dose-dependent reductions in meth self-administration in both sexes, although the pattern of effects differed. In males, reductions were most evident at lower meth doses, likely reflecting higher baseline responding at these doses and a greater dynamic range for detecting pharmacological effects. In contrast, females exhibited a more robust and consistent sensitivity to PPL-138 across conditions. Specifically, PPL-138 decreased meth self-administration, reduced meth choice, and significantly lowered both component-wise and cumulative consumption. These findings suggest that PPL-138 not only decreases meth intake but may also shift relative reinforcement away from drug toward the alternative (food) reinforcer in females. This capacity to reduce drug choice in the presence of a competing reward is a key feature of translational relevance, as it has produced concordant results with both human laboratory drug self-administration studies and clinical trials [39,40]. In contrast, although a trend toward reduced meth preference was observed in males, PPL-138 did not significantly alter choice behavior in this group. This highlights the importance of using choice-based procedures, as decreases in intake alone may not reflect meaningful therapeutic effects on decision-making processes underlying addiction. Compounds that reduce consumption without altering choice may have limited clinical utility [40], particularly in environments where alternative rewards are available.

In contrast to PPL-138, PPL-143 showed comparatively modest effects. In males, PPL-143 produced only trend-level reductions in meth self-administration and no effect on meth choice, although it did significantly reduce meth consumption at 0.1 and 0.3 mg/kg. This dissociation suggests that while PPL-143 may attenuate total drug intake, it does not meaningfully alter the motivational allocation between drug and nondrug rewards. In females, PPL-143 was even less effective. It failed to significantly reduce self-administration of meth or choice, despite decreasing cumulative consumption at the highest dose tested (3 mg/kg). Together, these findings indicate that PPL-143 has limited impact on the reinforcing efficacy of meth, particularly when animals are given a choice. Notably, the lack of enhanced efficacy despite greater NOP receptor engagement suggests that increasing NOP signaling, within the context of the NOP/mu receptor activity balance, may be insufficient to drive robust behavioral effects in this paradigm. The differential response to PPL-143 between males and females is noteworthy and aligns with evidence indicating that the N/OFQ-NOP receptor system exhibits pronounced sex-specific effects, with distinct roles in stress, pain, and affective processes across sexes. For example, genetic or pharmacological disruption of NOP receptor signaling can produce protective effects against stress-induced pain and anxiety in male rodents, whereas females often show persistent or unaltered responses, suggesting reduced sensitivity to NOP modulation [41,42]. These sex-dependent effects likely reflect underlying neurobiological differences, including hormonal influences, variations in dopaminergic and stress-related signaling, and differential valuation of drug versus nondrug rewards [39,43,44].

The reinstatement studies further differentiate the pharmacological profiles of the two compounds. PPL-138 effectively attenuated meth-primed reinstatement, indicating a capacity to reduce relapse triggered by re-exposure to the drug. This is a desirable property for pharmacotherapies targeting relapse [37,45]. However, this effect did not generalize to cue-induced reinstatement. Unexpectedly, PPL-138 increased drug-seeking behavior elicited by drug-associated cues, particularly at higher doses. Although surprising, this effect is reminiscent of prior findings from studies evaluating PPL-138 in models of cue-induced reinstatement of alcohol seeking [46]. A similar pattern was observed with PPL-143, which failed to significantly reduce meth-primed reinstatement and also enhanced cue-induced reinstatement. This dissociation between drug- and cue-induced relapse is highly informative, as these two forms of reinstatement are mediated by partially distinct neural circuits. Drug-primed reinstatement is more closely associated with dopaminergic mechanisms, whereas cue-induced reinstatement involves cortical and amygdala circuitry related to learned associations and incentive salience [47,48,49]. The potentiation of cue-induced reinstatement by both compounds suggests that, despite reducing ongoing drug intake, they may increase sensitivity to drug-associated stimuli. This represents a potential limitation for clinical translation, as cue-induced craving is a major driver of relapse in humans [50]. In contrast, PPL-138 reduced both meth intake and drug-primed reinstatement, indicating that the rewarding effects of meth are attenuated in the presence of this compound and thereby highlighting its therapeutic potential. Collectively, these findings underscore the importance of evaluating multiple relapse modalities when assessing candidate therapeutics.

The locomotor data further complicate the interpretation of PPL-138’s effects. Both PPL-138 alone and in combination with meth increased locomotor activity, with the combined treatment producing a supra-additive effect. This suggests that PPL-138 may enhance stimulant-related neural activity, possibly via dopaminergic or other excitatory pathways. Such effects could contribute to the increased cue-induced reinstatement observed, as heightened arousal or incentive salience attribution could amplify responses to drug-associated cues. Importantly, the increase in locomotor activity indicates that the reductions in meth self-administration are unlikely to be due to nonspecific motor suppression. Instead, PPL-138 appears to selectively reduce drug-taking behavior while simultaneously enhancing certain stimulant-like responses, pointing to a complex pharmacological profile.

The present findings are consistent with previous reports showing that buprenorphine reduces meth self-administration and meth-primed reinstatement while having little effect on cue-induced reinstatement [51]. The similarity between buprenorphine and PPL-138 suggests that maintaining a mixed mu opioid/NOP receptor profile may be sufficient to attenuate meth reinforcement and drug-primed relapse, but not cue-driven drug seeking.

Some limitations in this study should be acknowledged. First, the reinstatement studies were conducted only in females, limiting conclusions about sex differences in relapse-related effects. Second, the mechanisms underlying the divergent effects on drug-taking versus cue-induced seeking remain unclear and warrant further investigation. Finally, the locomotor-stimulant interaction suggests potential off-target or circuit-level effects that need to be better characterized.

In conclusion, these results support mixed mu opioid/NOP partial agonists as a promising pharmacological approach for MUD. Among the compounds tested, PPL-138 demonstrated the strongest efficacy, producing consistent reductions in meth reinforcement and meth-primed reinstatement, particularly in females. By contrast, PPL-143 showed comparatively limited efficacy despite greater NOP receptor activity, suggesting that further enhancement of NOP signaling does not necessarily improve therapeutic outcomes within this class of compounds. Importantly, neither compound reduced cue-induced reinstatement, and both enhanced cue-evoked drug seeking, emphasizing the need to evaluate multiple relapse modalities during medication development. Together, these findings indicate that distinct neurobehavioral mechanisms underlie meth taking and cue-driven relapse and support the continued use of choice procedures alongside relapse models when evaluating candidate treatments for MUD.

## 4. Materials and Methods

*Animals*. Sprague–Dawley rats were used throughout the study. Male and female animals were obtained from Charles River Laboratories (Raleigh, NC, USA) and weighed 180–200 g upon arrival. Rats were housed in pairs in self-standing plastic cages and maintained on a reverse 12 h light/dark cycle (lights off at 7:00 a.m.). All experimental procedures were conducted during the dark phase. Animals were allowed a 7-day acclimation period with ad libitum access to water and standard chow (5L0D PicoLab^®^ Laboratory Rodent Diet, Purina, Richmond, IN, USA). Prior to the start of experiments, rats were handled on three separate occasions. Housing conditions remained consistent throughout the study. All procedures were approved by the Institutional Animal Care and Use Committee at Florida Atlantic University.

*Drugs*. PPL-138 hydrochloride (14β-phenylpropanoyl-17-cyclopropylmethyl-7,8-dihydronoroxymorphinone) and PPL-143 hydrochloride ((5α)-14-(phenylpropanoyloxy)-17-(cyclopropylmethyl)-4,5-epoxy-3-hydroxy-morphinan) [35] were provided by Dr. Stephen M. Husbands, University of Bath (Bath, UK), and dissolved in vehicle containing 3% DMSO and 97% hydroxypropyl cellulose (0.5% in distilled water). Meth hydrochloride was obtained from the National Institute on Drug Abuse (NIDA) Drug Supply Program and was dissolved in 0.9% saline with PH adjusted to ~7.0 using 0.2 M sodium hydroxide. Food pellets (45 mg) for self-administration were obtained from Test Diet (5-TUM, Richmond, IN, USA). Buprenorphine Sustained Release was purchased from Wedgewood Pharmacy (Swedesboro, NJ, USA). Ceftriaxone (1 g vial), gentamicin (1 mg/mL) and heparin (1000 USP Units/mL) were obtained from FAU Comparative Medicine.

*I.v. catheter implantation*. Catheterization was performed as previously described [28,52]. Following surgery, rats were subcutaneously injected with a sustained-release formulation of buprenorphine at a dose of 0.6 mg/kg [53] and i.v. infused (0.3 mL) with ceftriaxone (200 mg/mL). For the duration of the experiments, catheters were flushed daily with 0.1–0.2 mL of 0.9% saline solution containing heparin and gentamicin.

*Equipment*. Self-administration experiments began one week after surgery and were conducted in operant conditioning chambers (Med Associates, Inc., St. Albans, VT, USA). Each chamber was equipped with two retractable levers mounted on the front panel, with a food pellet magazine positioned between them. Chambers also included auditory stimuli delivered via a speaker and visual cues provided by cue lights located above each lever. Drug infusions were delivered using syringe pumps (Med Associates, Inc.) and liquid swivels (Instech Laboratories, Plymouth Meeting, PA, USA), connected to plastic tubing encased in a flexible metal sheath for attachment to the external catheter connector. During the drug-versus-food choice procedure, responses on one (right) lever activated the infusion pump containing meth, whereas responses on the alternate lever resulted in delivery of a food pellet. In single drug self-administration sessions, responses on the right (active) lever activated the infusion pump, while responses on the other lever had no programmed consequences. Pump activation resulted in reinforcement delivery, except during extinction and reinstatement sessions, when responses no longer produced reinforcement. A computer-controlled system managed reinforcement delivery, stimulus presentation, and behavioral data acquisition.

*Meth-vs-food choice paradigm*. A drug-vs-food choice self-administration model was used [54]. The experiment was conducted in rats trained to lever press to receive either drug or food rewards in two-lever operant boxes. Rats were initially trained to lever press one lever (the right one) to self-administer meth (0.1 mg/kg/infusion) under an FR-1 schedule for 2 h across 3 days. Each lever press was accompanied by the illumination of a cue light placed above the lever that stayed on for 5 s, a time-out period in which, even if the rat pressed the lever, methamphetamine was not delivered. FR-1 was then increased to FR-3 for an additional 3 days. FR-3 was then increased to FR-5 for an additional 3 days. The same rats were then trained to lever press the other lever (the left one) for 45 mg food pellets. Each lever press on the left lever was accompanied by a tone that turned on for 5 s, a timeout period in which, even if the rat pressed the lever, food was not delivered. At this point, rats were trained to lever press for both levers. One lever was paired with intravenous meth infusions; the other was paired with the delivery of food pellets. Meth infusions and food pellets were offered on a FR-5 schedule of reinforcement (5 consecutive lever presses on the appropriate lever delivered 1 drug or food reward) in daily 2 h sessions. If an animal did not complete 5 consecutive lever pressings in one lever, the lever reset, and the reinforcement was not delivered. After this FR-5 meth/FR-5 food training phase, the animals were moved to another training phase where 4 meth doses in increasing order (0.025, 0.05, 0.1 and 0.25 mg/kg/inf) were presented in 2 h sessions. Rats were allowed to lever press for each meth dose for 29 min; thus, one session encompassed 4 29 min components. While one of the levers only delivered meth at various doses, the other simultaneously delivered food. A 1 min house light signaled the beginning of the various components. The effects of PPL-138 and PPL-143 at 0.1, 0.3 and 1.0 mg/kg injected by subcutaneous (sc) route were compared against their vehicle according to a counterbalanced within-subject design. Testing days were performed 4 days apart. In between testing days, sessions with escalating meth doses were conducted. For this experiment, a total of 28 rats (14 males and 14 females) were used. Seven rats per sex were assigned to testing with PPL-138 and seven to testing with PPL-143. Drug testing was conducted in parallel for both sexes. Experiments in males were performed first.

*Cue-induced reinstatement of meth seeking.* Female rats (n = 9) were trained to self-administer meth (0.05 mg/kg/infusion) under a fixed-ratio 1 (FR-1) schedule for 14, 2 h daily sessions conducted five days per week. Responses on the active lever resulted in a meth infusion paired with a 20 s presentation of a cue light located above the lever and a 20 s auditory tone (60–70 dB), both serving as conditioned stimuli. Following self-administration training, the same animals underwent extinction (EXT) training. During EXT, lever presses no longer produced meth infusions or associated cue presentations (light and tone). EXT sessions were conducted for 16 consecutive days, each lasting 60 min. Reinstatement testing was conducted in 60 min sessions during which presentation of the light–tone cue (but not meth) was contingent upon an active lever press. Reinstatement sessions were conducted every third or fourth day for a total of nine sessions and were interleaved with standard 1 h extinction sessions. The first reinstatement session served as a baseline test. In subsequent sessions, animals received either PPL-138 (0.1, 0.3, or 1 mg/kg, sc) or the vehicle, followed by PPL-143 (0.1, 0.3, or 1 mg/kg, sc) or the vehicle in a counterbalanced order according to a within-subject Latin square design, 20 min prior to each reinstatement session. The primary dependent measure was the total number of responses on the previously active (meth-paired) lever. Responses on the inactive lever were recorded throughout all experimental phases.

*Meth prime-induced reinstatement of drug seeking*. Additional female rats (n = 9) were trained to self-administer meth (0.05 mg/kg/infusion) under a FR-1 schedule during 2 h daily sessions conducted five days per week for 14 sessions. Responses on the active lever resulted in a meth infusion paired with a 20 s presentation of a cue light located above the lever. Following self-administration training, the same animals underwent EXT training. EXT sessions were identical to self-administration sessions, except that meth was no longer available, and continued for 20 consecutive sessions. After the final EXT session, a reinstatement session serving as a baseline test was conducted. Rats received an intraperitoneal injection of meth (0.5 mg/kg) 15 min prior to the session, which was carried out under the same conditions as EXT. Following three additional days of EXT, rats received pretreatment with PPL-138 (0.1, 0.3, or 1 mg/kg, sc) or the vehicle, followed by PPL-143 (0.1, 0.3, or 1 mg/kg, sc) or the vehicle, 20 min before intraperitoneal (ip) administration of meth (0.5 mg/kg). Sixty-minute prime-induced reinstatement sessions were conducted every third or fourth day, with PPL-138 and PPL-143 administered according to a within-subject Latin square design. EXT sessions were conducted between reinstatement tests. The primary outcome measure was the total number of responses on the lever previously associated with drug delivery. Responses on the inactive lever were also recorded throughout the experiment.

*Open Field test.* Assessment of locomotor activity in the open field was carried out as we have described previously [37,45,55]. The open field apparatus consisted of a square, open-top arena (50 cm wide × 30 cm high) painted black and dimly illuminated. The experiment began with a baseline trial in which female rats (n = 9) were allowed to freely explore the arena for 10 min. This was followed by four additional 10 min test sessions in which animals received a sc injection of PPL-138 (1 mg/kg) or the vehicle 20 min prior to ip injection of meth (0.5 mg/kg) or saline 15 min before testing. Treatments were administered in a counterbalanced order according to a Latin square design, with sessions conducted every three days. All sessions were video recorded using a camera mounted above the apparatus and analyzed with an automated tracking system (Ethovision^®^ XT, version 8; Noldus Information Technology, Wageningen, The Netherlands). Total distance traveled was the primary readout.

*Data analysis.* Tibco Statistica Version 13.5.0.17 was used for data analysis. In the analysis of the meth-versus-food choice self-administration procedure, meth rewards per component, meth choice per component, and meth consumption per component were evaluated by means of two-way repeated-measures ANOVA, with component and PPL compound doses used as within-subject factors. Cumulative meth consumption was analyzed using a one-way repeated-measures ANOVA. When ANOVA assumptions were not satisfied due to insufficient within-group variance, data were transformed into AUC values, and overall AUC was analyzed accordingly. Successful reinstatement was analyzed by means of paired *t*-tests. Effects of PPL-138 and PPL-143 on reinstatement and locomotor activity were analyzed using one-way repeated-measures ANOVA, with drug treatment as the within-subject factor. The level of significance was set at *p* < 0.05. Where appropriate, ANOVAs were followed by Tukey’s post hoc test.

## Data Availability

Study data will be provided upon request.
